# Sex- and Gender-Related Differences in Obesity: From Pathophysiological Mechanisms to Clinical Implications

**DOI:** 10.3390/ijms25137342

**Published:** 2024-07-04

**Authors:** Andrijana Koceva, Rok Herman, Andrej Janez, Matej Rakusa, Mojca Jensterle

**Affiliations:** 1 Department of Endocrinology and Diabetology, University Medical Center Maribor, 2000 Maribor, Slovenia; andrijana_koceva@yahoo.com; 2 Faculty of Medicine, University of Maribor, 2000 Maribor, Slovenia; 3 Department of Endocrinology, Diabetes and Metabolic Diseases, University Medical Center Ljubljana, 1000 Ljubljana, Slovenia; 4 Faculty of Medicine, University of Ljubljana, 1000 Ljubljana, Slovenia

**Keywords:** obesity, sex-related differences, gender-related differences, weight loss

## Abstract

Obesity, primarily characterized by excessive fat accumulation, is a multifactorial chronic disease with an increasing global prevalence. Despite the well-documented epidemiology and significant advances in understanding its pathophysiology and clinical implications, the impact of sex is typically overlooked in obesity research. Worldwide, women have a higher likelihood to become obese compared to men. Although women are offered weight loss interventions more often and at earlier stages than men, they are more vulnerable to psychopathology. Men, on the other hand, are less likely to pursue weight loss intervention and are more susceptible to the metabolic implications of obesity. In this narrative review, we comprehensively explored sex- and gender-specific differences in the development of obesity, focusing on a variety of biological variables, such as body composition, fat distribution and energy partitioning, the impact of sex steroid hormones and gut microbiota diversity, chromosomal and genetic variables, and behavioural and sociocultural variables influencing obesity development in men and women. Sex differences in obesity-related comorbidities and varying effectiveness of different weight loss interventions are also extensively discussed.

## 1. Introduction

Obesity is defined as excessive fat accumulation that can have a negative impact on health [1]. Body mass index (BMI) is a commonly used parameter for obesity classification. Adults with a BMI greater or equal to 25 kg/m^2^ are considered overweight, and a BMI greater or equal to 30 kg/m^2^ is considered obese. Obesity has become an ongoing pandemic as its prevalence in adults has more than doubled since 1990 [1]. The global prevalence of overweight and obese in the year 2020 was estimated at 2603 million (38%) and 988 million (14%) people, respectively. The prevalence of obesity in males (14%) was lower than in females (18%). Sex-specific differences are evident in all regions of the world [2]. Data from 3663 population-based studies with 222 million participants from 197 countries covering more than 99% of the world’s population demonstrated that the global age-standardised prevalence of obesity has increased from 8.8% in 1990 to 18.5% in 2022 in women and from 4.8% in 1990 to 14.0% in 2022 in men [3]. The age-standardized prevalence of obesity in adults increased from 1990 to 2022 in 188 countries for women and in all except one country for men, leading to a more than 20% increase in 49 countries for women and 24 countries for men [3]. The largest increases in obesity in women were in some countries in sub-Saharan Africa. In men, obesity increased most in the USA, Brunei, and some countries in central Europe, Polynesia, and Micronesia. In some countries in the Caribbean, the Middle East, and north Africa, obesity increased in both sexes. The countries with the largest absolute numbers of adults with obesity in 2022 were the USA, China, and India [3]. In low-income countries, obesity is more common in urban areas among wealthier subjects, especially women. In high-income countries, both sexes are equally affected. However, there is a greater impact on groups of lower socioeconomic status [4]. The National Health and Nutrition Examination Survey (NHANES), conducted between 2017–2018, showed a higher prevalence of obesity in non-Hispanic black women than men, and no sex differences in other races. There were also significantly more women with severe obesity (BMI ≥ 40 kg/m^2^) [5].

World Obesity Atlas predicts obesity trends by the year 2035 by assessing published data from 1975 to 2016 [2]. In the future, the prevalence of obesity is forecasted to increase. An additional increase was evident after the COVID-19 pandemic in the years from 2020 to 2022 [2]. Restrictions of movement, troublesome access to medical care, and changes in diet and behaviour are few of the reasons for the worsening of obesity epidemics in that period [2]. According to predictions for the year 2035, prevalence will almost double. Projected numbers of overweight and obese are 4005 million (51%) and 1914 million (24%) people, respectively. Globally, obesity will still be more common in females (27%) than males (23%). Regionally, trends will be the same, except for European and the Western Pacific regions, where obesity in males will surpass obesity in females [2].

The epidemiology, pathophysiology, and clinical implications of obesity have been well documented [1,6]. Nevertheless, a neglected factor in earlier research is the impact of sex, as there has traditionally been a significant lack of female representation in pre-clinical and clinical research [7,8]. In 2016, with increasing awareness of the importance of this biological variable, the United States National Institutes of Health (NIH) accepted a policy requiring future researchers to include males and females in every experiment funded by the NIH, which has led to increasing reports of sex differences in a variety of research fields [8], including obesity. In this narrative review, we comprehensively summarize evidence of sex- and gender-specific differences in obesity pathogenesis, risk factors, and clinical implications. Table 1 summarises the main subsections and topic highlights.

## 2. Sex and Gender-Specific Differences Underlying Obesity Pathology

### 2.1. Differential Body Composition and Fat Distribution in Men and Women

**Figure 1:**
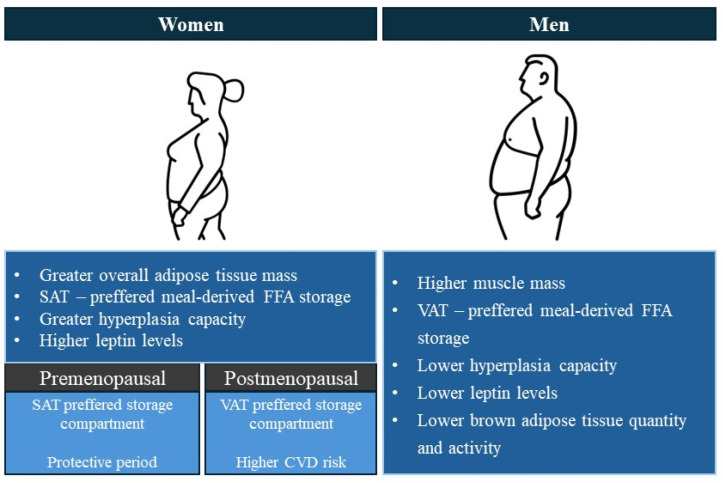
Differential body composition and fat distribution in women and men.

The main mechanism for obesity development is chronically increased energy intake and/or decreased energy expenditure [9]. The excess energy is stored in the adipose tissue with white adipose tissue (WAT) being the metabolic and endocrine organ that is the primary site of excess energy storage [9]. In times of positive energy balance WAT expands either by hypertrophy with an increase in cell size or by hyperplasia with an increase in cell number. The type of adipose tissue expansion differs depending on the depot and sex. Adipocyte hypertrophy is characteristic of the abdominal depot which is the main fat storage location in males, while adipocyte hyperplasia is more characteristic of the gluteofemoral depot which is the main fat storage location for females [10]. For the same BMI, the percentage of total adipose tissue in females is approximately 10% greater compared to males [11,12]. Although females have higher total body fat compared to males, males have higher visceral adipose tissue (VAT) [13]. The accumulation of fat derived from free fatty acids (FFA) and triglycerides is rate-limited by lipoprotein lipase (LPL) activity, which is higher in gluteal/subcutaneous fat depot in females and higher in the abdominal/visceral fat depot in males [14]. Therefore, females tend to store meal-derived FFA in the subcutaneous adipose tissue (SAT) in contrast to males, who preferably store meal-derived FFA in VAT [11,15]. Additionally, in premenopausal women, SAT has an increased ratio of alpha (α) 2 to beta (β) 1-2 adrenergic receptors, which leads to a decreased catecholamine—stimulated lipolysis compared to men and postmenopausal women. In premenopausal women, VAT has a reversed balance of adrenergic receptors, thus favouring lipolysis [16]. In times of positive energy balance, lower body SAT in females has a greater capacity for expansion by hyperplasia and is less responsive to catecholamine-stimulated lipolysis in contrast to the upper body fat [15]. Despite being a buffer for fat storage in periods of positive energy balance, increased gluteofemoral SAT has also been associated with a lower level of arterial calcifications and stiffness as well as improved lipid levels, implying atherosclerosis protection [17]. This sex-specific fat distribution is likely also connected to improved glucose metabolism and protection against diabetes development in premenopausal women, while preferential fat accumulation in the abdominal VAT in men is associated with an increased risk of metabolic disease [16]. A schematic highlighting the differential body composition and fat distribution in females and males is presented in Figure 1.

On the other hand, brown adipose tissue (BAT) and the browning of white adipose tissue are crucial for energy expenditure functions [18]. Browning or beiging of adipocytes refers to the process by which WAT acquires characteristics similar to BAT, including increased mitochondrial content and the expression of uncoupling protein 1 (UCP1) [19]. Increasing BAT activation and browning of WAT can influence energy homeostasis and protect against obesity and obesity-related metabolic diseases [20]. BAT and the process of browning white adipocytes exhibit significant sexual dimorphism, potentially contributing to differences in obesity and metabolic disease risks between men and women. However, sex and gender differences in human thermogenic adipose tissue are still understudied. Studies have demonstrated that women generally have higher amounts of BAT and exhibit greater BAT activity through non-shivering thermogenesis compared to men [21,22,23]. This heightened activity is due to several factors, including hormonal influences and intrinsic cellular properties. Studies show that sex hormones regulate BAT activity in a sex-specific manner through direct and indirect mechanisms. Oestrogens induce a stimulatory effect on BAT activity, while androgens appear to have an inhibitory effect. The crosstalk between sex hormones and glucocorticoids adds to the mechanisms that control sexually dimorphic BAT activity [10,21]. In addition, women have more inducible beige adipocytes, within their subcutaneous fat depots compared to men. This is supported by evidence showing higher expression levels of genes related to mitochondrial function and thermogenesis in female adipocytes [24]. Furthermore, women display greater sensitivity to cold exposure, which enhances the browning process [24]. Studies have also suggested that oestradiol could regulate thermogenic adipose tissue at the central level via hypothalamic AMPK activation [25]. It is important to note that despite understanding the higher prevalence, mass, and activity of thermogenic adipose tissue and the initial clues of the physiological mechanisms behind these differences, the consequences for metabolic disease progression are still largely unknown beyond speculation [23].

Adipokines, such as leptin and adiponectin, secreted from adipose tissue also have a significant association in obesity pathogenesis and its metabolic complication [26].

Leptin is a peptide hormone and a well-known adipokine which interacts with leptin receptors in the hypothalamus and brainstem [27]. Leptin levels are in direct proportion to total fat content, and high levels inhibit food intake and increase lipolysis, thermogenesis, and energy expenditure [27]. Leptin levels have a higher correlation to SAT and thus are higher in females, and these differences seem to be more pronounced during puberty and decrease after menopause [10,27]. Since these sex-specific differences in adiposity appear at the onset of puberty and decrease after menopause in females or with declining testosterone in males, one can conclude that sex steroid hormones have a substantial impact on obesity development [10]. Adiponectin concentration is negatively connected to visceral adipose tissue content, and this finding is stronger in females compared to males [13]. This sexual dimorphism may be due to higher adiponectin mRNA expression in ectopic fat in females. A study by Christen et al. using MRI for quantification of abdominal SAT and VAT and measuring leptin and adiponectin levels showed that adiponectin levels are higher in females than in males, which is not completely explained by differences in VAT. On the other hand, leptin was also higher in females and was strongly related to total body fat [13]. Obesity is associated with higher leptin levels, lower adiponectin levels, and a higher leptin/adiponectin (L/A) ratio [28]. According to a study of 2258 children with overweight/obesity, girls with higher L/A ratios were more likely to have insulin resistance, glucose impairment, and dyslipidemia, while boys were more likely to have insulin resistance [28].

Some organokines, such as chemerin and betatrophin, have a role in glucose and lipid metabolism [29,30]. Chemerin acting through CMKLR1 is adipokine, the expression of which is influenced by metabolic status [29]. Chemerin levels correlate with BMI and waist-to-hip ratio and increase during development of impaired glucose tolerance or diabetes. Animal studies show that chemerin levels are increased in male compared to female rats at the pre-pubertal stage, and chemerin expression was attenuated in ovariectomized female rats but enhanced in orchiectomized male rats [29]. Its receptor, CMKLR1, on the other hand, showed no difference in its expression after gonadectomy but was markedly influenced by nutritional status, as its levels markedly decreased with high fat diet (HFD) [29]. Betatrophin acts as a hepatokine involved in triglyceride hepatic metabolism [30]. Betatrophin levels have a strong correlation with atherogenic lipoprotein subfraction. Betatrophin levels are higher in all subjects with type 2 diabetes or obesity and in females, but not in males, indicating a possible influence of oestrogen on betatrophin-mediated lipoprotein metabolism [30].

### 2.2. Differential Energy Expenditure in Men and Women

Daily energy expenditure is comprised of three components: activity-induced energy expenditure, diet-induced expenditure, and resting energy expenditure. Resting energy expenditure is determined by body size and composition and is the main component of the total energy expenditure [31]. Diet-induced energy expenditure for a mixed diet results in 10% of daily energy expenditure [32]. Physical activity-induced energy expenditure is defined by body size and movement and it is the most variable component of daily energy expenditure [33]. Activity-induced energy expenditure changes during different life stages as it increases from 20% at age 1 to around 35% at age 18 and decreases after age 50 [33]. The largest changes in body composition occur during adolescent growth, emphasizing the importance of physical activity from a young age. Physical activity is a determinant of peak fat-free mass, which is essential for preventing sarcopenic obesity later in life [31].

Data on 4240 individuals, out of which 1870 were twin pairs, explored the transition period from adolescence to young adulthood and showed that physical inactivity during adolescence significantly increased the risk of obesity in adulthood, especially central obesity [34]. In a monozygotic twin substudy, co-twins with obesity in adulthood were found to be less physically active in adolescence, and discordant monozygotic twins showed that obesity development was independent of genetic effects. Additionally physical activity also declined after developing obesity [34]. With increasing age, there is a decrease in physical activity levels, decrease in fat-free mass, and an increase in fat mass [33]. For males, changes in body composition during exercise training are highly related to initial fat mass, as subjects with higher initial fat mass lose more fat than leaner subjects. This was not seen in females [33]. Combining aerobic training with resistance exercises has been linked to decreased visceral fat in males and subcutaneous leg fat in females [35]. While physical activity intensity has been linked to fat loss in both sexes, physical activity duration has been linked to fat loss in males only [36].

There are clear sex differences in energy partitioning between males and females. In times of increased energy requirements, females oxidize a greater proportion of FFA, while males have a higher preference for using carbohydrates, thus increasing glycogen utilization. Postprandially or in resting conditions, females—in contrast to males—oxidize less FFA and tend to store FFA in adipose tissue [15,37]. Estradiol is responsible for reducing resting/postprandial lipid utilization by suppressing hepatic lipid oxidation while promoting skeletal muscle fatty acid oxidation during exercise and in postmenopausal females, overall lipid utilization is decreased [38]. Despite relying on lipid utilization during exercise, females are less likely to have a reduction in fat mass in resting conditions [15]. The strategy of lipid utilization during periods of prolonged exercise or food shortage provides females with a higher ability to spare protein mass which is a clear survival advantage. However, during times of food abundance, this survival advantage becomes a predisposition for obesity development [15].

### 2.3. Sex-Specific Roles of Sex Steroid Hormones

#### 2.3.1. Lessons from Animal Models

Oestrogen acts on the central nervous system, particularly on the hypothalamus, which regulates body weight homeostasis and energy balance by controlling food intake and energy expenditure [38]. By activating oestrogen receptor (ER)α, prominently expressed on pro-opiomelanocortin (POMC) neurons within the arcuate nucleus, oestrogen modulates and suppresses food intake. Additionally by activating ERα in neurons of the ventromedial hypothalamus oestrogen also stimulates physical activity and thermogenesis and regulates fat distribution [38]. In adipose tissue, oestrogens can act on oestrogen receptors alpha (ERα) or oestrogen receptors beta (ERβ). Animal studies demonstrated that the distribution of these receptors is particularly important for oestrogen’s ability to modulate its effects on fat distribution [16,39,40]. ERα knockout mice had increased adiposity and visceral fat accumulation [40]. In ovariectomized mice [39], oestrogen substitution reduced visceral fat mass in wild-type and ERβ knockout mice, but not in ERα knockout mice, which indicates that the lipolytic effects of oestrogens are primarily mediated by ERα. The ERβ knockout mice had a greater reduction in visceral fat mass, which can suggest that ERβ activation may oppose oestrogen’s lipolytic effects mediated by ERα activation [16,39,41]. Males have a relative lack of ERα in the visceral depot, which can explain the preferential storage of fat in the visceral compartment [16,40]. Animal studies showed that despite female mice having a lesser propensity to obesity development when exposed to HFD than their male counterparts, the natural protective effect from HFD-induced obesity diminishes after ovariectomy, a type of surgical menopause [42]. Some also hypothesized that follicle-stimulating hormone (FSH) may also play a role in menopause-induced obesity, as blocking FSH interaction with its receptor by an administration of FSH antibodies protected both male and female mice against HFD-induced obesity by inducing the browning of WAT, thermogenesis, and increasing energy expenditure [43].

Testosterone is an androgen receptor (AR) agonist. AR distribution and expression are higher in VAT compared to SAT [44]. Testosterone can act on the AR or can also be converted to dihydrotestosterone (DHT) or oestradiol by 5α-reductase and aromatase, respectively. Dihydrotestosterone is a more potent androgen that cannot be aromatized to oestradiol [45]. A recent study by Sebo and Rodeheffer assessed the effect of testosterone deficiency and the individual contributions of testosterone, oestradiol, and dihydrotestosterone to body composition, total adiposity, and fat distribution by using mouse models with castration-induced hypogonadism that were fed a standard or HFD [45]. Hypogonadal mice gained more fat mass and less lean mass compared to controls on both diets, with the rate of fat gain being more pronounced in the HFD group, indicating that hypogonadism accelerates obesity development when dietary fat intake is high. Hypogonadism altered body composition but not fat distribution [45]. To evaluate the individual-specific contributions of different hormones on body composition and fat distribution, the castrated hypogonadal mice on HFD underwent hormone replacement therapy either with testosterone, oestradiol or DHT, and the gonadally intact mice were treated with aromatase inhibitors [45]. Testosterone replacement successfully reduced HFD-induced fat expansion in both visceral and subcutaneous depots. Oestradiol replacement only modestly reduced HFD-induced fat expansion in the visceral depot with no significant effect on subcutaneous fat, while DHT replacement had reduction only in subcutaneous fat with no significant reduction in total fat mass compared to controls. These findings suggest that testosterone replacement has an anti-obesogenic effect in a hypogonadal state [45]. The gonadally intact mice treated with aromatase inhibitors gained more fat, with a preferential increase in visceral fat accumulation compared to controls [45]. Since aromatase inhibitor therapy leads to a decrease in testosterone conversion to oestradiol, this gain in fat mass also implies that the anti-obesogenic effect of testosterone in males may be at least partly mediated by its conversion to oestradiol [45].

Testosterone also appears to be important for metabolic programming [46,47]. Studies on animal models showed that testosterone exposure and neonatal androgenization in female mice led to an increase in WAT with adipocyte hypertrophy, a decreased insulin-dependent lipogenesis, hypoadiponectinemia, and a decrease of the intensity of POMC neuronal projections within the arcuate nucleus. These changes led to increased food intake and hyperleptinemia with reduced ability to up-regulate POMC expression [46,47,48].

#### 2.3.2. Insights from Human Physiology

Over the last 15 years, new insights into the links between sex steroid hormones and diet, eating behaviour, neuroendocrine control of energy homeostasis, and obesity have greatly expanded our knowledge of sex-specific human physiology of obesity. In premenopausal females, there is a higher ERα protein content in the abdominal than the gluteal depot, and ERβ protein is higher in the gluteal than the abdominal depot [49]. This higher ERα/β ratio in the visceral depot limits adipose fat accumulation, and a lower ERα/β ratio in the gluteal fat leads to preferential fat accumulation and storage in that depot [16]. Additionally, oestradiol also upregulates the number of the anti-lipolytic α-2a adrenergic receptors in SAT but not in VAT, leading to a diminished epinephrine-mediated lipolytic effect in SAT, which additionally shifts fat accumulation from VAT to SAT [50].

Given the complex relationship of these hormones in the etiopathogenesis of obesity, alterations in sex hormones are associated with various changes in body composition, fat distribution, and metabolic diseases. While all females gain SAT over time, with the decrease in oestrogen throughout menopause, energy expenditure and fat oxidation in females also decrease, which eventually leads to an increase in total body fat as well as VAT [51]. Thus, menopause, evident from a decrease in oestradiol levels and an increase in FSH, triggers a relative shift in adipose tissue accumulation, specifically from SAT to VAT accumulation, and this finding is partially reversed by oestrogen hormone replacement therapy [52]. Low oestrogen levels in menopause or premature ovarian insufficiency are also connected to increased risk of diabetes development [53], and hormonal replacement therapy seems to reduce this risk and improve glucose homeostasis [54,55].

There is a bidirectional relationship between obesity and testosterone. In a sex-specific, two-sample Mendelian randomization analysis, Loh et al. provided evidence of this bidirectional interplay by showing that in females, high bioavailable testosterone can be both a cause as well as consequence of obesity, while in males higher bioavailable testosterone was associated with lower hip circumference and lower fasting glucose and obesity was linked to lower bioavailable testosterone and higher oestradiol [56]. Subjects with prostate cancer treated with androgen deprivation therapy, on the other hand, have increased visceral abdominal fat and subcutaneous fat and a decrease in lean mass as a consequence of testosterone deficiency [57]. Age-related testosterone decline is also associated with a change in body composition, leading to an increase in fat mass and decrease in fat-free mass [58]. The effects of acute testosterone deficiency were studied by Santosa et al. in a prospective randomized trial, in which they found that acute testosterone withdrawal resulted in a greater meal-derived FFA storage in SAT by increasing LPL and acyl coenzyme A synthetase activity. Therefore, testosterone in males may have a tonic role in femoral fat storage by supressing LPL activity [59].

Conversely, obesity can also induce hypogonadism. While obesity-related secondary hypogonadism is a well-known condition in males, there is evidence suggesting the existence of obesity-related secondary hypogonadism in females as well [60]. Male obesity-related hypogonadism is characterized by higher conversion of testosterone to oestradiol in adipocytes, which then exerts negative feedback on gonadotropin-releasing hormone (GnRH) secretion. This, in turn, leads to a decreased production of luteinizing hormone (LH) and FSH and therefore impaired testicular testosterone production [60,61]. Female-obesity-related hypogonadism is characterized by increased endogenous LH clearance and/or reduced pituitary response to GnRH. This results in reduced LH levels as well as LH pulse amplitudes, which in turn impairs ovarian function [60]. As opposed to low testosterone levels in male-obesity-induced hypogonadism, females with obesity may have increased androgen production regardless of the presence of PCOS, which further reduces LH levels and predisposes them to metabolic dysfunction [60]. High androgen levels in females, like in polycystic ovary syndrome (PCOS), promote insulin resistance and alterations in glucose tolerance [62].

In a prospective study, Huang et al. demonstrated sex differences in the association of maternal androgen levels with the development of metabolic syndrome in the offspring later in life [63]. By following sons and daughters for up to 50 years after birth they concluded that although sons had significantly worse cardiometabolic profiles compared to daughters, higher prenatal levels of androgens in the mothers were associated with an increased metabolic syndrome incidence in the daughters but not in sons, further confirming the effect of perinatal androgen exposure on metabolism programming [63]. To conclude, masculinized energy metabolism in daughters born to mothers with higher levels of androgens seems to be a predisposition to visceral adiposity and insulin resistance later in life.

Another example of the implication of sex steroid hormones in body composition, fat distribution, and obesity development is seen with gender-affirming hormone therapy (GAHT) [64,65]. Here transgender women are usually treated with oestrogens and anti-androgens, while transgender men are treated with testosterone [64]. A study with a baseline obesity prevalence of 25% in the transfeminine group and 39% in the transmasculine group found that even though obesity rates increased in both groups during hormone therapy, specifically within 2–4 months in the transmasculine group and after 22 months in the transfeminine group, the rates of obesity and weight gain were higher in the transmasculine group (with testosterone being the most likely reason) [64]. A recent review analysed 20 studies focusing on body composition changes in transgender individuals receiving GAHT. Oestrogen therapy in transgender women was associated with a decrease in lean mass and waist-to-hip ratio and an increase in fat mass and BMI, while testosterone therapy in transgender man was associated with an increase in lean mass, increase in BMI and waist-to-hip ratio with a tendency to gain visceral adiposity and lose subcutaneous adiposity [65], again confirming the impact of sex hormones on fat distribution and body composition.

**Figure 2:**
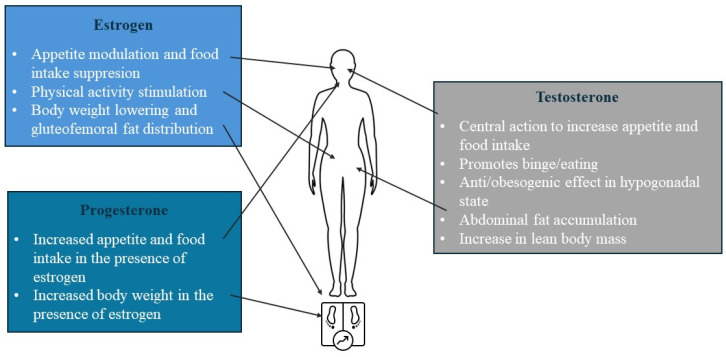
Summarised effect of sex steroid hormones on appetite.

In addition to their importance in regulating fat distribution, sex hormones are also involved in appetite regulation, as oestrogen decreases appetite and food intake in women, while progestine, progesterone in the presence of oestrogen, and testosterone increase appetite and food intake [66]. This is evident from the varying caloric intake across the menstrual cycle. Increasing oestrogen levels cause a progressive decrease in eating during the follicular [67] and the 4-day periovulatory phase, when estradiol is at its highest levels, which is not seen in anovulatory women [68,69]. Additionally, resting energy expenditure fluctuates during the menstrual cycle, reaching its lowest point during the early follicular phase and increases to approximately 50–100 kcal/day more in the luteal phase [67]. The effect of sex steroid hormones on appetite is summarised in Figure 2.

### 2.4. Sex Difference in Obesity-Related Genetic Susceptibility

It is well known that around 40–70% of BMI variability can be attributed to genetic factors, and studies have identified around 900 genetic loci associated with BMI, which explains only 6% of the population variance in adult BMI [70]. Some studies additionally confirmed sexual dimorphism in the genetic loci for some anthropometric traits with a stronger effect in females than in males [71,72].

Sex chromosomes are an additional important factor for sex dimorphism in obesity. This has been proven by the development and use of the four-core genotype (FCG) mouse model, a model that separates the contributions of gonads and sex chromosomes to obesity development [73]. Through production of XX and XY mice with ovaries and XX and XY mice with testes and analysis of all four genotypes, one can differentiate whether a specific trait is influenced by chromosomal sex (XX or XY) or gonadal sex (ovaries or testes) [73]. Chen et al. gonadectomized the FCG mice to diminish the hormonal effects on adiposity and showed that gonadectomized XX-carrying mice, regardless of the gonadal type before gonadectomy, had worse HFD-induced obesity outcomes with greater food intake, increased adiposity, and accelerated weight gain and also developed fatty liver, hyperleptinemia, hyperinsulinemia, and hyperlipidemia [74]. When studying different chromosomal combinations, such as XO and XXY, the negative effects of a HFD on obesity development were more pronounced by increasing the number of X chromosomes and not by the absence of the Y chromosome, confirming the importance of the X chromosome on obesity development and its sexual dimorphism [74]. In another study, the group identified a positive relationship between Kdm5c gene expression and adiposity [75]. Kdm5c is an X chromosome gene, which influences gene expression involved in extracellular matrix reorganization, which is crucial for adipose tissue expansion. Kdm5c expression in females is higher, likely because of X chromosome inactivation escape and experimental reduction of Kdm5c dosage in XX females to a level usually present in XY males leads to reduced adipose tissue expansion [75].

Genome-wide association studies (GWAS) also showed that there is strong sexual dimorphism in the genetic regulation of WAT. Bernabeu et al. investigated the effect of sex on genetic architecture by analysing 530 complex traits in 450,000 individuals in the UK Biobank [76]. When considering the autosomal genome, they found that the trait with the largest number of sex-dimorphic single nucleotide polymorphisms (SNPs) was the waist-to-hip circumference ratio, with a total of 2421 sex-dimorphic SNPs, representing 100 unique loci [76]. Kilpeläinen et al. identified a locus near the *IRS1* gene that was associated with reduced body fat percentage, more specifically with less SAT compared to VAT in males but not in females, and this was also linked to an unfavourable lipide profile, insulin resistance, decreased levels of adiponectin, and increased risk for diabetes and coronary artery disease [77]. In another recent study, Kaisinger et al. undertook an exome-wide association study by using data from 419,692 UK Biobank participants and identified genes for which rare heterozygous loss-of-function is associated with an increased BMI in females (*DIDO1*, *PTPRG*, and *SLC12A5*) and in males (*SLTM*), with effect sizes up to 8 kg/m^2^ [70]. A common limiting factor of these studies is that the analysis is restricted to individuals of European ancestry.

### 2.5. Sex-Related Differences in Gut Microbiota

The gut microbiome has a vital role in digestion, metabolism, and nutrient absorption. Additionally, it has other critical functions, such as preserving the intestinal immune system response and tolerance, as well as stimulating and regulating hormone synthesis [78]. Dysbiosis can be defined as a decrease in gut microbiota compositional variety and/or functional diversity. Although it is not known whether dysbiosis is the cause or the consequence of a certain disease, it is well known that significant changes in the gut microbiota were noticed in people with obesity. One of the most researched parameters since its discovery in 2005 is the Firmicutes:Bacteroidates (F:B) ratio [78]. Animal and human studies showed an increase in the F:B ratio, which is associated with a greater capacity to extract energy from the diet, in obesity [79,80,81] and a decrease in the F:B ratio with weight loss [79], although this hypothesis has been challenged by underpowered studies. Gut microbiota composition and diversity are also affected by sex. An observational study that compared microbiota diversity in males and females with high sex hormone levels to those with low sex hormone levels showed that high sex steroid hormone levels are associated with greater microbiota diversity in both sexes, and females with higher oestrogen levels had a higher abundance of Bacteroidetes and lower levels of Firmicutes phyla compared to those with low oestrogen levels [82]. Qin et al. showed that female mice on HFD showed slower weight gain as well as slower increase in F:B ratio compared to male mice fed the same diet, once again showing that females are more resistant to diet-inducing obesity [83]. He et al. also showed that there are gut microbiota sex differences as a response to probiotic strain Lactobacillus reuteri, with a positive effect on immune response, an increase in the abundance of phylum Bacteroidates, and a decrease in Firmicutes phyla seen in females and not in males [84]. Firmicutes phyla are the main producers of butyrate, which is an anti-inflammatory metabolite. The higher prevalence of Firmicutes phyla in males with obesity can lead to an increased generation of butyrate, which may suppress the immune response [9]. Bacteroidetes phyla—which are more prevalent in females with obesity, on the other hand—are gram-negative bacteria containing lipopolysaccharides (LPS) in the membrane, which are associated with a more robust immune response [9]. A stronger immune response may be the reason that delays the development of obesity-related metabolic diseases in females [9].

Additionally, the gut microbiota is involved in sex steroid metabolism [85,86,87]. Oestrogen metabolism occurs mainly in the liver through a two-stage reaction by hydroxylation and conjugation, and conjugated oestrogens are later excreted in the bile and passed into the intestine in conjugated form. Here, gut microbial beta-glucuronidase plays a vital role in regulating physiological oestrogen metabolism, as this enzyme deconjugates oestrogens in the intestine. These oestrogens regain biological activity and can be reabsorbed [85]. This reabsorption of deconjugated oestrogens in the blood and the liver is called enterohepatic recycling of oestrogens, a process which is important for maintaining sex hormone homeostasis. Increased beta-glucuronidase activity is inversely correlated with faecal oestrogens [87], and by increasing the production and activity of beta-glucuronidase there is an increase in enterohepatic circulation of biologically active oestrogens [85]. Gut microbiota, by having a role in the deconjugation of DHT and testosterone, may be an important regulator of intestinal androgen metabolism as well [86]. A study showed that mice with normal microbiota composition had remarkably increased free levels of the most potent androgen, DHT, while mice lacking gut microbiota had substantially lower levels of free DHT [86]. This finding was also tested in human physiology, as young men also had substantially increased faecal free DHT levels and the group proposed that diet changes affecting gut microbiota composition or probiotic use might modulate intestinal androgen metabolism [86].

## 3. Sex and Gender—Specific Risk Factors Influencing Overweight/Obesity Pathology

### 3.1. Behavioural Neuroadaptive Food Intake Preferences

Obesity is associated with structural and functional changes in the brains reward system [88,89]. Palatable food consumption activates the mesolimbic dopaminergic pathway, inducing rewarding effects. Repeated activation of this pathway by palatable food consumption in obesity leads to neuroadaptation in its downstream inhibitory cortico-striatal circuits, which leads to compulsive behaviour and loss of control in food intake [89,90,91]. Although this neuroadaptation is shared in both sexes, females are more susceptible to obesity-induced neural adaptations in the reward regions than males [88]. Based on a systematic review of functional neuroimaging studies, females showed greater neural responses in striatal/limbic system and frontal/cortical system in response to food cues and may be more sensitive to visual food cues, although this conclusion was made based on a limited number of studies with small sample sizes [88,92]. Furthermore, studies show that females compared to males experience stronger tonic and trait food cravings overall [93,94,95] and may be less successful in regulating and suppressing cue-induced food cravings [96,97].

There appear to be gender differences in food preferences and eating behaviours between males and females [98,99,100,101,102]. A cross-sectional study in 21 European countries showed that females have greater consumption of fruits and vegetables compared to males [98,99]. A study examining comfort food preferences across gender showed that females were more likely to prefer sweet tastes and snack-based comfort food, such as candy or chocolate, while males were more likely to choose meal-based comfort food, like pizza or pasta [100]. Another cross-sectional study by Lombardo et al. looking at gender differences in taste and food habits in 2021 subjects showed that females preferred more whole grain foods, cereals, and vegetables, while males preferred more eggs, meats, and processed meat. While males ate faster and skipped breakfast more often, females were more likely to eat uncontrollably, even when not hungry [101]. Feraco et al. used an online survey to examine gender differences in food preferences and eating habits in 2198 subjects and also confirmed that males are more likely to prefer red and processed meat, while females aligned with healthier food choices and were more likely to choose vegetables, whole grains, tofu, and dark chocolate. Males also ate faster and dined out, while females more frequently ate without hunger [102].

### 3.2. Sociocultural Role Modelling and Gender-Different Psychological Influence during Development

As proposed according to the gender intensification hypothesis in 1983 by Hill and Lynch [103], as adolescents develop physically and emotionally, they tend to align themselves closely with the stereotypes associated with their gender. The main stereotype in males is mesomorphism, and that in females is physical attractiveness, which in Western societies is closely linked to thinness [104]. With the increase in adiposity following puberty, the discrepancy between the actual and ideal body is increased, creating a period of vulnerability for girls to body dissatisfaction [104] as well as for the development of eating disordered behaviours, such as binge-eating disorder or night-eating syndrome [105,106].

Females generally experience greater levels of body dissatisfaction in comparison to males, likely due to more significant societal pressure and pressure from friends and family to adhere to cultural standards, being more influenced by social media to follow appearance ideals and face a higher amount of criticism on their physical appearance from both male and female peers compared to male individuals [104,107,108]. A recent study by Dougherty et al. examined the relationship between interpersonal stress and shape/weight concerns in a group of boys and girls with overweight/obesity using ecological momentary assessment [109]. Interpersonal stress was connected to shape/weight concerns in girls with overweight/obesity but was not related to shape/weight concerns in boys with overweight/obesity. Additionally, girls who experienced higher feelings of loneliness, social rejection, and a desire for more friends had higher levels of shape/weight concerns as well [109].

Obesity is strongly associated with a wide range of psychological and mental disorders, from low self-esteem and body image disturbance to depression, eating disorders, and low quality of life [110]. The connection between obesity and depression seems to be bidirectional, as some studies show that antidepressant use increases the risk of obesity, while others indicate that obesity is often the cause of depression. Depression is more common in females with obesity than males [111,112]. Eating-disordered behaviours, such as binge-eating disorder and night-eating syndrome, are also commonly found in people with obesity. Female sex can be a risk factor, as these disorders are more common in females [110,113]. Obesity also has an impact on quality of life separately from depression and low self-esteem, including impacts on education opportunities, income, and relationships. Even here, we can find gender differences, as female gender was associated with a lower chance to complete an advanced educational program, lower household income, and lower likelihood to get married [110].

In summary, although food preferences and eating behaviours may vary between males and females, females are more susceptible to obesity-induced neural adaptations and less successful in managing food cravings. Furthermore, females generally experience greater body dissatisfaction—often due to societal pressure, social media influence, and criticism from peers—as well as more eating-disordered behaviour and depression.

## 4. Clinical Implications of Sex-Related Differences in Obesity

### 4.1. Differential Sex and Gender Risks of Obesity-Related Comorbidities

Obesity has also sex-specific differences in related comorbidities. Even though obesity is more prevalent in females, females may be protected from obesity-associated metabolic implications [114,115]. Studies on animal models showed that male rodents compared to females exhibit an earlier onset of obesity, a higher degree of obesity, and have a greater prevalence of accompanying risk factors such as hyperglycaemia, hyperinsulinemia, and hypertension when subjected to an HFD [9,114,115]. Obesity is an important risk factor for diabetes development [116] and according to a meta-analysis by Guh et al. diabetes incidence rate ratios are higher in females than males [117]. On the other hand, there is also a sex difference in the time of diabetes development in people with obesity, as studies show that females are diagnosed with diabetes mellitus at a later age and have a higher BMI at diagnosis compared to males [118,119,120]. These differences are likely due to the impact of sex steroid hormones on body composition, fat distribution, and energy metabolism and are attenuated with increased age and menopause [121]. The major proposed underlying mechanism of obesity-induced hypertension is the activation of the sympathetic nervous system, however, obesity seems to increase sympathetic nerve activity (SNA) in males but not in females [122]. A potential explanation might be in the different central actions of insulin and possibly leptin in patients with obesity [122]. Specifically, insulin increases SNA similarly in lean males and females, while this sympathoexcitatory response to insulin is amplified in males with obesity, it is abolished in females with obesity [123]. In addition angiotensinogen expression and secretion is greater in visceral adipocytes, and studies show that VAT also has greater SNA compared to SAT adding to the development of sex differences [124]. Waist circumference and BMI are also strongly associated with obstructive sleep apnoea (OSA). In a cross-sectional study including 14,3326 females and 22,896 males, OSA was diagnosed in 6.4% of females and 13.8% of males. OSA prevalence increased with increasing BMI, and among individuals with morbid obesity (BMI ≥ 40 kg/m^2^), OSA was present in 50% of males compared to 30% of females [125].

Evidence from epidemiological studies shows that obesity is not only associated with an increased risk of cancer but also an increased risk of recurrence and cancer-related mortality in several malignancies [126]. According to the report from the International Agency for Research on Cancer Working Group on Body Fatness in 2016, there is sufficient evidence for an association between obesity and cancer risk in 13 cancers, such as postmenopausal breast cancer, endometrial cancer, ovarian cancer, colorectal cancer, oesophageal cancer, stomach cancer, pancreatic cancer, cancer of the gallbladder, liver cancer, kidney cancer, meningioma, multiple myeloma, and thyroid cancer [126]. Similar findings were found in an umbrella review by Kyrgiou et al. [127]. There is a sex variation in the relative risk of cancer development. When looking at the total number of obesity-related cancers, females had a higher number of obesity-related cancer cases [126]. The higher overall incidence among females is likely due to the contribution of endometrial, ovarian, and postmenopausal breast cancer, and obesity-related cancers that concern both sexes show a higher incidence in males [128].

To summarise, all of this shows that while males may be more at risk for the development of the metabolic implications of obesity, females are more at risk for the psychological implications of obesity. While obesity-related cancer seems to be more prevalent in females, this is mostly due to gynaecological malignancies, and when looking at cancers that concern both sexes, there is a higher incidence in males.

### 4.2. Gender-Differential Effectiveness of Weight Loss Interventions

#### 4.2.1. Non-Pharmacological Interventions

There seem to be gender differences in treatment-seeking, with females being more likely to pursue a lifestyle weight loss intervention [121] as well as more likely to be offered a weight loss intervention [129]. A study analysing differences in healthcare professional counselling of individuals with overweight and obesity found that females were more likely to receive physician counselling to control/lose weight, reduce fat/calorie intake, and increase physical activity compared to males [129]. This could be the result of healthcare providers perceiving females as being more receptive than males or because females visit healthcare providers more often, thus having a greater chance to develop a trusting relationship and comfort in discussing sensitive subjects such as weight loss. On the other hand, while females were more likely to adopt recommendations to increase physical activity, there were no significant gender differences in reported efforts to control/lose weight or reduce fat/calorie intake, meaning that both genders were similarly susceptible to behavioural change if offered proper counselling [129].

Regarding diet success, a recent meta-analysis of sex differences in weight loss on a low-carbohydrate diet (LCD) showed that while most included studies did not report a significant difference in weight loss on a LCD, some of them had a numerical difference, with a greater absolute weight loss in males over females [130]. When a significant sex difference was reported, all but one included study showed that males lost more weight on LCD compared to females [130], which has also been a conclusion in previous analyses on sex differences in weight loss intervention effectiveness [131].

#### 4.2.2. Pharmacological Interventions

At the moment there are six medications for non-syndromic obesity: orlistat, naltrexone/bupropion, liraglutide, semaglutide, and tirzepatide—which are all approved by the Food and Drug Administration (FDA) and European Medical Agency (EMA)—and phentermine/topiramate, which is only approved by the FDA [132]. An analysis of 26,522 patients who were prescribed antiobesogenic medications between 2015 and 2016 revealed that pharmacotherapy was more often prescribed to females. Specifically, 85.5% of naltrexone/bupropion, 82.2% of phentermine/topiramate, and 72.4% of liraglutide prescriptions were given to females [133]. Furthermore, when looking at the demographics of the randomized patients, the majority of studies on the efficacy of antiobesogenic medications have involved a larger cohort of female participants, leading to the underrepresentation of males [132]. The STEP clinical program examining semaglutide efficacy in treating obesity showed clear female predominance, with STEP 1 including 74.1% of females [134], STEP 3 including 81% of females [135], STEP 4 including 79% [136], and STEP 5 including 77.6% of females [137]. STEP 2 recruiting people with obesity and diabetes was more gender-balanced, including 49% of male participants [138]. The latest SURMOUNT program evaluating the efficacy of the GIP/GLP1-RA tirzepatide included a greater proportion of males (30–49%) compared to other trials, likely due to capping female enrolment at 70% [139].

This female predominance and male underrepresentation are particularly important since some studies show sex-specific differences in pharmacotherapy efficacy. A study conducting exposure–response analysis using liraglutide for obesity treatment showed a clear positive relationship between liraglutide exposure and weight loss [140]. Females also had a 32% higher drug exposure than males of similar body weight and achieved greater weight loss at any given exposure compared to males [140]. They also found that the exposure–weight loss response did not plateau in males receiving liraglutide at a dose of 3 mg, possibly suggesting that additional weight loss may be achieved by using doses above 3 mg, which has not been clinically tested [140]. Subgroup analyses of the STEP program also showed a greater weight reduction in females compared to males. Possible reasons could be exposure differences, as females may have a lower mean body weights, leading to higher exposure to GLP1-RA, different feeding behaviour regulation due to the impact of sex hormones, slower gastric emptying in premenopausal females, and higher frequency of gastrointestinal side effects in females [141]. Preregistration clinical trials of the combination naltrexone/bupropion and phentermine/topiramate also showed a tendency of higher weight loss in females than males [142].

To conclude, females are more likely to be offered a weight loss intervention, including pharmacotherapy which is evident by the male underrepresentation in most registration trials of anti-obesogenic medication as well as the lower prescription of obesity pharmacotherapy in males. Studies on pharmacotherapy show a tendency for greater efficacy in females and more research is needed to examine whether there is a need for sex-tailored dosing of pharmacotherapy.

#### 4.2.3. Bariatric Surgery

There seems to be gender disparity in bariatric surgery utilization among eligible patients, with females comprising over 80% of patients and males being underrepresented in most bariatric surgery studies [143]. This can be a consequence of gender-based differences in perception of body weight impacting motivation to pursue surgical treatment as well as possible gender bias in patient selection by the physicians [143]. A study examining patients’ motivation for seeking surgical treatment showed that when the primary motivation for surgical treatment was appearance and embarrassment, the subjects were far more likely female, younger, and with a lower presenting BMI. When the primary motivation was stated as a medical condition and health concern, the subjects were more likely to be male, older, and with fewer depression symptoms and better mental health [144]. Another study by Kochkodan et al. looked at the sex and gender differences in preoperative characteristics, postoperative complications, and comorbidity resolution in patients undergoing bariatric surgery [145]. Out of the total 61,708 patients, 78% were female and 22% were male. While both females and males benefited from surgical treatment, preoperatively, males had significantly more risk factors, a higher BMI and comorbidity burden, and postoperatively had increased surgical complications, less weight loss, and less comorbidity resolution [145]. Females, on the other hand, continued to express lower body image and lower psychological well-being scores, leading to lower postoperative satisfaction despite having lower preoperative BMI, having lost more body weight, and having greater comorbidity resolution postoperatively, showing again that body image perception has a higher impact in females, who may perceive obesity differently than males [145]. This shows that there is a gender inequality in the offering of bariatric surgery to males, and when offered, it is performed at a later stage of the disease. Although females have better surgical outcomes, there is a need for increased attention to the psychological effect of surgery, as they may benefit from psychological treatment pre- and postoperatively.

While many have implied that there is a beneficial effect of bariatric surgery and weight loss on cancer risk [128,146,147,148], one study also implied that bariatric surgery may be more effective in lowering cancer risk in females in contrast to males [149], although this has been limited by the relatively short mean time of follow-up and the smaller sample size of male participants [146,149].

## 5. Challenges and Future Directions

Even though great progress has been achieved in the understanding of the impact of sex in obesity pathophysiology, past obesity research has often been gender-neutral or with female underrepresentation and recent clinical registration trials of anti-obesity medications show male underrepresentation. A sex-specific analysis is often missing. Given the emerging trend toward personalized medicine and patient-tailored approach, considering the implication of sex and gender on different stages of obesity development is crucial in optimizing both its prevention and treatment effectiveness. Further obesity research is also needed on the full spectrum of gender identities to ensure awareness, inclusiveness, and reflectiveness of the diverse needs of the population.

## 6. Conclusions

There is a clear sexual dimorphism in obesity prevalence, which can be explained by differences in obesity pathophysiology, including fat distribution, energy metabolism, and gut microbiota diversity, as well as the impact of chromosomes and genetic predisposition. Additionally, sex and gender differences in risk factors contribute to weight gain disparities. Obesity-induced comorbidities also show sexual dimorphism, with males more susceptible to the metabolic consequences and females more vulnerable to psychopathology. Regular obesity screening is mandatory for both sexes since studies show that obesity in males is often recognized and treated at a later stage, when obesity-related comorbidities are well developed. Understanding the factors that predict body dissatisfaction and psychological challenges is crucial for developing effective prevention programs. Further research on sex and gender-related differences across different treatment modalities is needed to ensure optimized and personalized obesity management.

## Figures and Tables

**Table 1:** Summary of variety of variables influencing sex and gender—specific differences in obesity.

Variables Influencing Sex and Gender Dimorphism in Obesity	
**Sex and Gender—Specific Differences underlying Obesity Pathology**	Body Composition and Fat Distribution Energy Expenditure Roles of Sex Steroid Hormones Genetic Susceptibility Gut Microbiota
**Sex and Gender—Specific Risk Factors Influencing Obesity Pathology**	Behavioural Neuroadaptive Food Intake Preferences Sociocultural Role Modelling and Gender—Specific Psychological Influence during Development
**Clinical Implications of Sex and Gender—Related Differences in Obesity**	Differential Risks of Obesity-Related Comorbidities Differential Effectiveness of Weight Loss Interventions
